# Clinical and Molecular Predictors of Response and Survival Following Venetoclax Plus Hypomethylating Agents in Relapsed/Refractory Acute Myeloid Leukemia: A Single-Center Study in Chinese Patients

**DOI:** 10.3390/cancers17040586

**Published:** 2025-02-08

**Authors:** Linya Wang, Haitao Gao, Qiang Fu, Qian Jiang, Hao Jiang, Yu Wang, Lanping Xu, Xiaohui Zhang, Xiaojun Huang, Feifei Tang

**Affiliations:** 1 Peking University People’s Hospital, Peking University Institute of Hematology, National Clinical Research Center for Hematologic Disease, Beijing Key Laboratory of Hematopoietic Stem Cell Transplantation, Peking University, Beijing 100044, China; 2 Peking-Tsinghua Center for Life Sciences, Academy for Advanced Interdisciplinary Studies, Peking University, Beijing 100871, China

**Keywords:** relapsed or refractory AML, venetoclax, hypomethylating agents, genetic characteristics

## Abstract

Purpose: This study aimed to investigate the efficacy and the clinical and molecular predictors of response and survival following venetoclax plus hypomethylating agents (VEN + HMAs) in adult relapsed/refractory acute myeloid leukemia (R/R AML) patients. Methods: We retrospectively analyzed 197 adult R/R AML patients who received the VEN + HMAs regimen. Molecular profiling was performed using targeted next-generation sequencing (NGS) of 139 genes to explore potential response and survival genetic predictors. Results: The median treatment cycle was 1 (1–4) cycle. The composite complete remission (CRc) rate, encompassing complete remission (CR) and CR with incomplete hematologic recovery (CRi), was 44.7%, while the overall response rate (ORR) reached 59.9%. With a median follow-up period of 14.0 months (range: 0.7–54.0 months), the 1-year and 2-year overall survival (OS) rates were 55.4% and 40.2%, respectively. Multivariate analyses revealed that mutations in *NPM1* and *SRSF2* were significantly associated with improved response rates. Conversely, prior exposure to HMA therapy, early relapse, and the presence of *GATA2* mutations were linked to lower response rates. Regarding survival outcomes, the *CBFB-MYH11* fusion gene, as well as mutations in *NPM1* and *IDH1/2*, were found to be favorable prognostic factors for OS, whereas mutations in *FLT3-ITD*, *TP53*, *DNMT3A*, and *GATA2* were associated with worse OS. Conclusions: The VEN + HMAs regimen demonstrated considerable efficacy in the treatment of R/R AML patients, with both response rates and overall survival being influenced by distinct genetic features. These findings provide valuable insights into optimizing personalized treatment strategies for this challenging patient population.

## Simple Summary

Relapsed or refractory acute myeloid leukemia (R/R AML) poses significant treatment challenges, necessitating novel therapeutic approaches. This study evaluated the effectiveness of venetoclax combined with hypomethylating agents (VEN + HMAs) as a potential treatment and examined genetic predictors of patient outcomes. The findings highlight the promising efficacy of VEN + HMAs and reveal that genetic mutations significantly influence treatment response and survival. These insights underscore the importance of genetic profiling in guiding personalized treatment strategies for R/R AML patients.

## 1. Introduction

Despite extensive clinical practices, the prognosis of relapsed/refractory acute myeloid leukemia (R/R AML) patients remains considerably poor, with 5-year overall survival (OS) rates of less than 10% [1,2]. No standard therapy has been established for treating R/R AML. Allogeneic hematopoietic stem cell transplantation (allo-HSCT) stands as the sole treatment with the potential to cure R/R AML [2]. Nevertheless, only a small fraction of patients are suitable candidates for allo-HSCT, mainly due to the inadequate response required for a successful transplantation. This highlights the pressing demand for new treatments that can improve the rates of response and survival in these patients.

The combination of Venetoclax (VEN) with hypomethylating agents (HMAs) has been broadly adopted for the treatment of newly diagnosed elderly or unfit adult patients with AML [3]. Given the promising results of this therapeutic approach in de novo unfit AML patients, where a VIALE-A study reported a composite complete remission (CRc) rate of 66.4% and an improved median overall survival (OS) of 14.7 months [4], it has increasingly been adopted for use in R/R AML cases. Previous studies showed various results following the VEN + HMAs regimen treating R/R AML patients, with response rates ranging from 33.3% to 51.0% [5,6,7,8,9]. Previous studies have indicated that mutations in *NPM1* and *IDH1/2* could predict a superior response to VEN plus HMAs [10,11,12], while mutations in *FLT3-ITD*, *TP53*, and *K/NRAS* were linked to lower response rates [12,13]. Large-sample reports on the treatment of R/R AML patients using VEN plus HMAs regimens are still relatively limited. In addition, R/R AML patients in previous studies did not entirely have next-generation sequencing (NGS) data [5,14]. The correlation between genetic characteristics and outcomes of VEN-based therapy in R/R AML remains unclear.

## 2. Materials and Methods

### 2.1. Data Source and Eligibility

**Figure 1:**
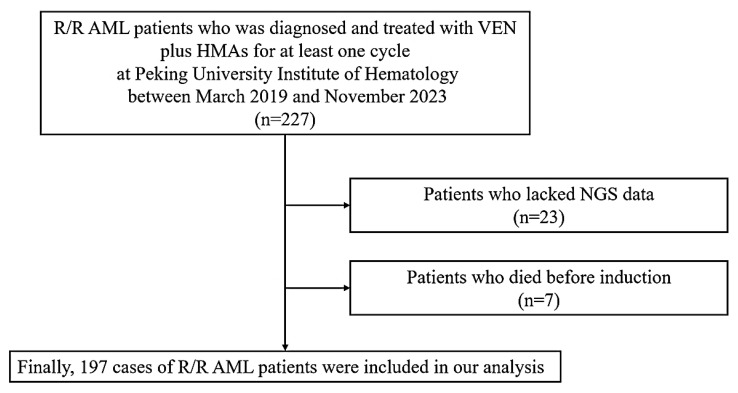
The outline of research methodology.

We reviewed the data of R/R AML patients who were diagnosed and treated with VEN plus HMAs for at least one cycle at Peking University Institute of Hematology between March 2019 and November 2023. Finally, 197 patients were included in this study. Patients who lacked NGS data (*n* = 23) or died before induction (*n* = 7) were excluded (Figure 1).

The diagnostic criteria for AML were based on the 2016 World Health Organization classification [15]. Genetic risk was classified according to the 2022 ELN risk stratification [16]. The definitions of refractory AML and relapsed AML were identical to the guidelines [16,17,18]. NGS was performed on all included patients using a targeted panel of 139 genes related to AML, myelodysplastic syndrome (MDS), and myeloproliferative neoplasms (MPN). Bone marrow samples were collected, and genomic DNA (gDNA) was extracted using the QIAamp DNA Blood Mini Kit (Qiagen, Hilden, Germany). Library preparation was conducted using the Illumina DNA Prep Kit (Illumina, San Diego, CA, USA), and sequencing was performed on the NovaSeq 6000 platform (Illumina, San Diego, CA, USA) with paired-end 150-bp reads (PE150). The panel covered hotspot regions for detecting single nucleotide variants (SNVs), insertions/deletions (indels), internal tandem duplications (ITDs), and partial tandem duplications (PTDs). The captured DNA fragments were sequenced with a median on-target sequencing depth of ≥2000× to ensure reliable detection of mutations. Variants were analyzed using a bioinformatics pipeline, including base calling, alignment, and variant annotation with ANNOVAR and internal tools. Filtering criteria required the read depth supporting mutations to be ≥30×, with balanced support from both positive and negative strands. The turnaround time from sample collection to the final report was approximately 7–10 days. The complete list of targeted genes is provided in Table S1.

Follow-up data were gathered from medical records, telephone calls, and visits to outpatient clinics, with the final follow-up recorded on 31 March 2024. Informed consent was secured from all participants. This study adhered to the principles of the Declaration of Helsinki and received approval from the ethics committee of Peking University People’s Hospital (2025PHB064) on 27 January 2025.

### 2.2. Induction Treatment

Induction treatment consisted of two regimens: VEN combined with azacytidine (AZA) [VEN + AZA] or VEN combined with decitabine (DAC) [VEN + DAC]. In the VEN + AZA regimen, VEN was administered orally, starting at 100 mg on day 1, 200 mg on day 2, and escalating to 400 mg from days 3 to 28, with adjustments made based on clinical needs. Additionally, if patients were concurrently using antifungal medications, the VEN dosage was 100 mg daily. AZA was given subcutaneously at a dose of 75 mg/m^2^ from days 1 to 7. In the VEN + DAC regimen, the VEN dosing schedule was identical to that of the VEN + AZA group, while DAC was administered intravenously at 20 mg/m^2^ from days 1 to 5.

### 2.3. Post-Induction Therapy

Subsequent treatment strategies should be carefully tailored to consolidate the remission and reduce the risk of relapse. Allo-HSCT is recommended for all patients, regardless of whether they achieve CRc after induction therapy. The decision to proceed with allo-HSCT is made based on a comprehensive assessment, including the patient’s remission status, clinical fitness, availability of donors, and the willingness of both the patient and family. For patients who are not candidates for allo-HSCT due to age, comorbidities, or lack of donor availability, maintenance therapy with VEN + HMAs can be considered to prolong remission.

### 2.4. Response Criteria

Bone marrow assessments (BMA) were routinely conducted on day 28 following the initiation of therapy. If the morphologic leukemia-free state (MLFS) was not achieved, BMA was repeated every 2–4 weeks to confirm response or reassess disease status. BM biopsy was generally performed at initial diagnosis and repeated when there was a change in AML status or clinical suspicion of disease progression. CR was characterized by an absolute neutrophil count exceeding 1000 cells per cubic millimeter. Platelet counts over 100,000 per cubic millimeter, independent from red-cell transfusions, and a BM blast presence of less than 5%. CRi met all CR criteria except for either neutropenia (absolute neutrophil count below 1000 cells per cubic millimeter) or thrombocytopenia (platelet count below 100,000 per cubic millimeter). MLFS is defined as a condition characterized by a BM blast presence of less than 5%, the absence of blasts with Auer rods, the absence of extramedullary disease, and no requirement for hematologic recovery. Partial remission (PR) was characterized by a BM blast percentage level of 5–25%, with a reduction of over 50%. Non-remission (NR) was defined as not achieving PR. CRc encompassed both CR and CRi. The overall response rate (ORR) included both CRc and PR. Measurable residual disease (MRD) was evaluated during each BMA using multiparametric flow cytometry (MFC) analysis and RT-PCR of BM aspirate samples. Any measurable level of MRD through MFC was considered positive. MRD was also tracked using RT-PCR for detectable genes [19].

### 2.5. Statistical Analysis

The baseline characteristics of the patients were presented using frequencies and percentages for categorical data and medians, along with ranges for continuous data. For comparing grouped variables, the chi-square test or Fisher’s exact test was applied, while continuous variables were assessed using the nonparametric Mann–Whitney U-test. Univariate and multivariate logistic regression analyses were conducted to identify prognostic factors affecting treatment response, and Cox regression was used to analyze survival outcomes. OS was calculated from cycle 1, day 1 of therapy, until death, or the time of the last follow-up and estimated using the Kaplan–Meier method. Survival distributions between groups were compared using the log-rank test. The median follow-up period was calculated using the reverse Kaplan–Meier method. All tests were two-sided at a significance level of 0.05. Statistical analyses were performed using R statistical software (version 4.3.1, http://www.r-project.org) and Empower Stats software (version 4.1, http://www.empowerstats.com, X & Y Solutions, Inc., Boston, MA, USA).

## 3. Results

### 3.1. Patient Characteristics

This retrospective study included 197 R/R AML patients with a median age of 47 years (range 18–83). Details of patient characteristics are provided in Table 1. Of these, 71 patients (36.0%) had primary refractory AML, while 126 (64.0%) had relapsed AML. Among 126 relapsed AML patients, 96 patients (76.2%) had previously undergone chemotherapy, and 30 patients (23.8%) relapsed after allo-HSCT. Certain patients had prior exposure to VEN or HMAs, with each patient receiving at least one of these agents or their combination during at least one phase of therapy. HMAs had been previously administered to 111 patients (56.1%). Among these, 72 patients received HMAs during the induction phase, 34 during the consolidation phase, and 58 during the maintenance phase. Fifty-eight patients (29.4%) had received prior VEN therapy, with 35 patients during the induction phase, 15 during the consolidation phase, and 27 during the maintenance phase.

According to the 2022 ELN risk stratification [16], 108 patients (54.8%) were classified in an adverse risk category. Based on cytogenetic analysis of 157 patients, 98 patients (62.4%) had adverse risk cytogenetics. Genetic data of all patients were included in the evaluation of treatment outcomes and the impact of genetics on treatment response.

### 3.2. Treatment Characteristics

The details of the treatment characteristics are displayed in Table 1. Of 197 R/R AML patients, 184 (93.4%) received the VEN + AZA regimen, while 13 (6.6%) were treated with VEN + DAC. Patients began treatment immediately after relapse was confirmed, although initial induction therapy may have involved alternative regimens. The median time from relapse to the initiation of VEN + HMA therapy was 21 days (range, 1–116). Notably, 80 patients with specific molecular mutations had previously received targeted molecular therapies. Of these, 72 out of 79 patients with *FLT3-ITD* mutation (91.1%) received *FLT3-ITD* inhibitors: 46 (63.9%) were treated with sorafenib, 22 (30.6%) with gilteritinib, and 4 patients (5.5%) sequentially received both. Moreover, 10 of 52 patients with *IDH1/2* mutation (19.2%) received *IDH1/2* inhibitors. Among overall R/R AML patients, 144 patients (73.1%) received a 28-day regimen of VEN therapy during the first cycle. Additionally, dose interruptions and reductions due to adverse events were reported in 53 patients (26.9%). Furthermore, 73 patients (37.1%) underwent subsequent allo-HSCT, with 65 of these patients (89.0%) receiving haploidentical HSCT (haplo-HSCT).

### 3.3. Response and OS

**Figure 2:**
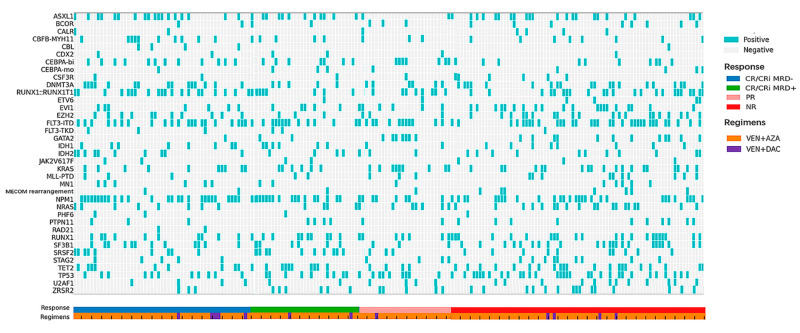
Genetic landscapes of 197 relapsed or refractory (R/R) acute myeloid leukemia (AML) patients. Oncoprint shows cytogenetic and mutational characteristics for all the R/R AML patients receiving venetoclax plus hypomethylating agents (VEN + HMAs). Patients are grouped by response and annotated with colored bars below the grid. The treatment regimens of each patient are annotated with colored bars below the response bar.

Treatment responses are detailed in Figure 2 and Table 2. After a median of one therapy cycle (range, 1–4), the ORR (19.8% CR, 24.9% CRi and 15.2% PR) was 59.9%.

**Figure 3:**
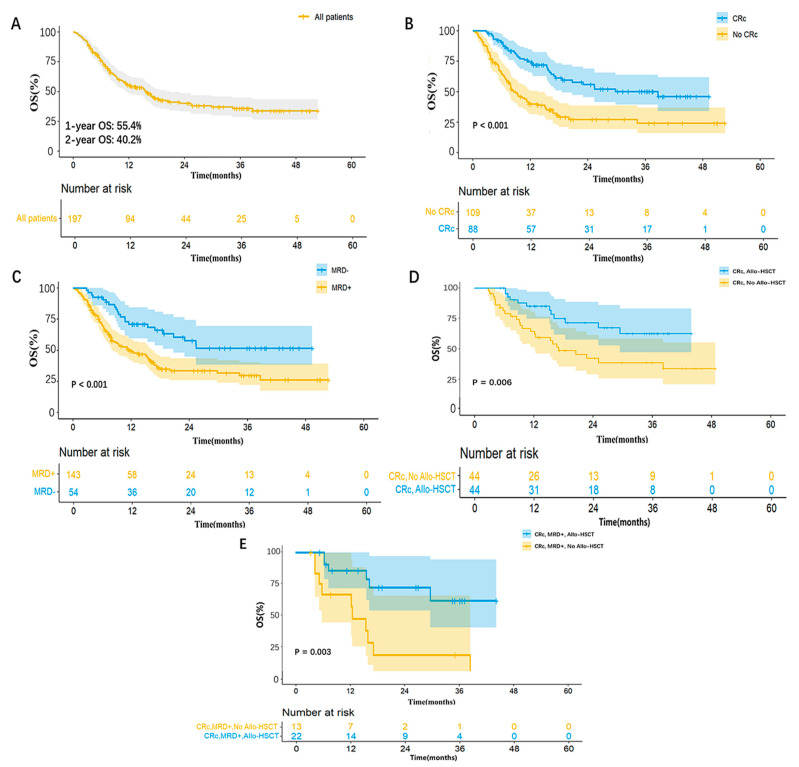
Overall survival (OS). (**A**), OS in all R/R AML patients (*n* = 197); (**B**), OS based on composite complete remission (CRc); (**C**), OS based on measurable residual disease (MRD) status after venetoclax plus hypomethylating agents (VEN + HMAs) therapy; (**D**), OS based on receiving allo-HSCT (Allogeneic hematopoietic stem cell transplantation) among patients achieving CRc after VEN + HMAs therapy; (**E**), OS based on receiving allo-HSCT among patients achieving CRc but MRD-positive after VEN + HMAs therapy.

During a median follow-up of 14.0 months (range: 0.7–54.0), 110 patients (55.8%) died, including 39 from relapse, 35 without achieving CRc, 14 from transplant-related causes, and 22 from other causes. In the total patient cohort, the median OS was 15.7 months (95% CI, 11.9–19.5; Figure 3A). Patients achieving CRc post-therapy had better prognoses compared to those without CRc [the median OS: not reached vs. 8.9 months (95% CI, 6.4–11.4); *p* < 0.001; Figure 3B]. OS analysis based on post-therapy MRD status showed MRD-negative patients had significantly better survival outcomes than MRD-positive patients [the median OS: not reached vs. 11.8 months (95% CI, 7.1–16.4); *p* < 0.001; Figure 3C].

Among patients achieving CRc after induction therapy, those who underwent allo-HSCT demonstrated better survival compared to those who did not undergo allo-HSCT [the median OS: not reached vs. 17.2 months (95% CI, 9.3–25.2); *p* = 0.006; Figure 3D]. Regardless of whether they underwent allo-HSCT, OS was comparable between MRD-negative patients (*p* = 0.15). In contrast, the MRD-positive patients who underwent allo-HSCT demonstrated significantly prolonged OS compared to those who did not undergo allo-HSCT [the median OS: not reached vs. 12.5 months (95% CI, 7.6–17.5); *p* = 0.003; Figure 3E].

### 3.4. Predictors of Response

All patients in our study have complete genetic data, and each patient harbored at least one mutation. The overall profile regarding genetic characteristics and treatment response is presented in Figure 2. Patients who were categorized in the adverse ELN risk group presented lower CRc rates compared to those in the non-adverse group (36.4% vs. 51.4%; *p* = 0.035). Patients with *NPM1* mutation showed higher CRc rates compared to those without *NPM1* mutation (56.8% vs. 36.2%, *p* = 0.004). Moreover, patients with *GATA2* and *K/NRAS* mutations showed lower CRc rates compared to those without these mutations (13.6% vs. 48.6%, *p* = 0.002; 36.0% vs. 50.0%, *p* = 0.047, respectively).

**Figure 4:**
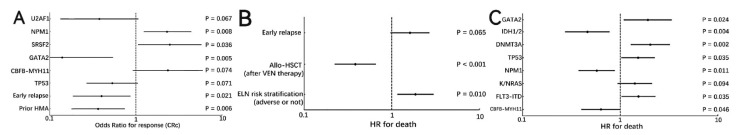
Multivariable analysis. (**A**) Predictors of response (composite complete remission [CRc]); (**B**) Clinical predictors of OS; (**C**) Genetic predictors of OS. Variables of *p* < 0.1 in univariable analysis (Tables S2 and S3) were included in this multivariable logistics model.

Univariable analysis for genetic predictors of response (CRc) is shown in Table S2. Multivariable analyses of response to VEN-based therapy are summarized in Figure 4A. Prior HMAs [Odds ratio (OR) 0.4, 95% CI, 0.2–0.7, *p* = 0.006] and early relapse (OR 0.4, 95% CI, 0.2–0.9, *p* = 0.021) were unfavorable factors for response. Mutations in *NPM1* (OR 2.3; 95% CI, 1.2–4.3; *p* = 0.008) and *SRSF2* (OR 2.5; 95% CI, 1.1–5.8; *p* = 0.036) were favorable factors for response. Mutation in *GATA2* (OR 0.1; 95% CI, 0.0–0.5; *p* = 0.005) was an unfavorable factor for response.

### 3.5. OS Predictors of Clinical Characteristics and Regimens

Univariable analysis revealed that the adverse ELN risk stratification, prior HMAs, and early relapse were risk factors for OS (Table S3). The cycles of VEN + HMAs > 2 and allo-HSCT after VEN therapy were protective factors (Table S3).

Multivariable analysis showed that allo-HSCT after VEN therapy [Hazard Ratio (HR) 0.4; 95% CI, 0.2–0.7; *p* < 0.001] was the protective factor for OS. Adverse ELN risk stratification [HR 1.9; 95% CI, 1.2–3.0; *p* = 0.010] was the risk factor for OS. (Figure 4B).

### 3.6. Molecular Predictors of OS

**Figure 5:**
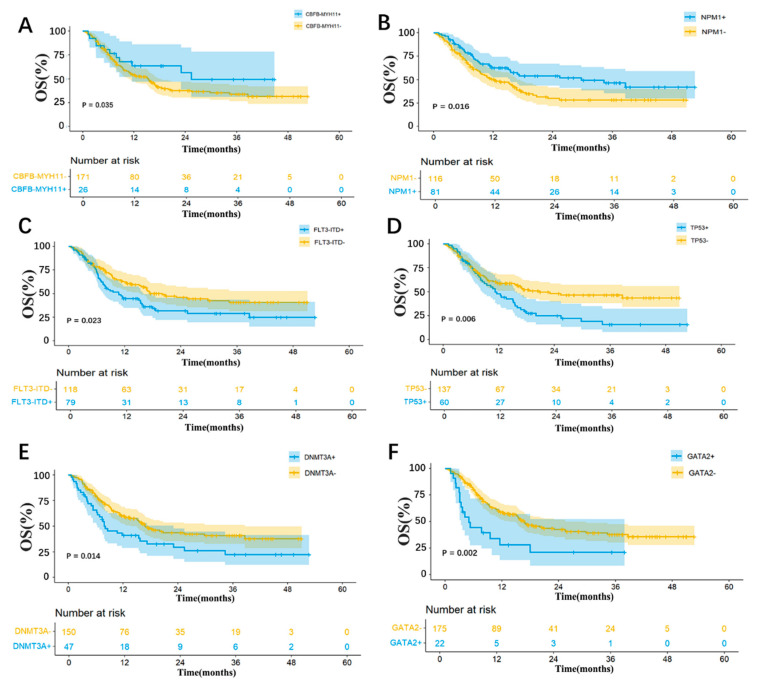
Genetic subgroup analysis of OS. (**A**), CBFB-MYH11 fusion gene; (**B**), NPM1 mutation; (**C**), FLT3-ITD mutation; (**D**), TP53 mutation; (**E**), DNMT3A mutation; (**F**), GATA2 mutation.

In our study, the R/R AML patients with the *CBFB-MYH11* fusion gene displayed a better prognosis compared to those without [the median OS: not reached vs. 15.4 months (95% CI, 11.3–19.4); *p* = 0.035; Figure 5A]. In addition, the OS of R/R AML patients receiving VEN + HMAs is closely associated with the following important molecular mutations. Favorably, the presence of *NPM1* mutation was associated with improved survival compared to patients lacking this mutation [the median OS: 29.8 months (95% CI, 10.4–49.2) vs. 12.3 months (95% CI, 7.2–17.4); *p* = 0.016; Figure 5B]. However, several molecular mutations are more closely related to poor prognosis. Patients with *FLT3-ITD* mutation had a shorter OS compared to those without this mutation [the median OS: 10.7 months (95% CI, 6.9–14.5) vs. 20.0 months (95% CI, 12.4–27.7); *p* = 0.023; Figure 5C]. *TP53* mutation was significantly associated with inferior OS compared to patients without this mutation [the median OS: 11.2 months (95% CI, 8.4–14.0) vs. 20.8 months (95% CI, 3.6–38.0); *p* = 0.006; Figure 5D]. The patients harboring *DNMT3A* mutation also showed a decreased OS in comparison to those without the mutation [the median OS: 7.9 months (95% CI, 4.4–11.3) vs. 16.9 months (95% CI, 13.0–20.8); *p* = 0.014; Figure 5E]. Those with *GATA2* mutation exhibited markedly inferior survival outcomes than those without the mutation [the median OS: 5.0 months (95% CI, 2.6–7.5) vs. 16.7 months (95% CI, 13.0–20.4); *p* = 0.002; Figure 5F].

Univariable analysis revealed that the *NUP98-NSD1* and *BCR-ABL* fusion genes, as well as mutations in genes such as *FLT3-ITD*, *K/NRAS*, *TP53*, *DNMT3A*, *SF3B1*, *EZH2*, and *GATA2*, were risk factors for OS (Table S3). The *CBFB-MYH11* fusion gene and mutations in genes such as *NPM1*, *IDH1/2*, and *SRSF2* were protective factors for OS (Table S3).

Multivariable analysis showed that mutations in *NPM1* [HR 0.6; 95% CI, 0.4–0.9; *p* = 0.011], *IDH1/2* [HR 0.5; 95% CI, 0.3–0.8; *p* = 0.004], and *CBFB-MYH11* fusion gene [HR 0.6; 95% CI, 0.4–1.0; *p* = 0.052] were protective factors for OS. Mutations in *FLT3-ITD* [HR 1.5; 95% CI, 1.0–2.3; *p* = 0.035], *TP53* [HR 1.5; 95% CI, 1.0–2.3; *p* = 0.035], *DNMT3A* [HR 2.0; 95% CI, 1.3–3.2; *p* = 0.002], and *GATA2* [HR 1.9; 95% CI, 1.1–3.4; *p* = 0.024] were the risk factors for OS (Figure 4C).

## 4. Discussion

To our knowledge, this is the most extensive retrospective study to date that analyzed clinical and molecular predictors of response and OS following VEN + HMAs in R/R AML [5,6,8,9,10,11,20,21]. In addition, unlike other previous studies, all the R/R AML patients in our study have NGS data [5,14,20]. This large-scale study indicates that VEN + HMA therapies are effective for patients with R/R AML and that molecular mutations may provide insights into the therapeutic response as well as OS.

In our study, the CRc rate of VEN + HMAs therapies for treating R/R AML patients was 44.7%. Specifically, the CRc rates for the VEN + AZA and VEN + DAC treatment groups were 43.5% and 61.5%, respectively (*p* = 0.21). Our study did not show a significant improvement in CRc rates compared to previous studies, which reported variations from 33.3% to 51.0% [5,6,7,8,9]. This may be attributed to the inclusion of a high proportion of patients with adverse molecular or clinical risk factors in our study: twenty-five post-transplant relapse patients (25/73, 34.2%), 79 patients (40.1%) with *FLT3-ITD* mutations, and 60 patients (30.5%) with *TP53* mutations. The proportion of these high-risk patients in our study is higher than that reported by Aldoss I. et al., in which 29.0% of post-transplant patients experienced relapses, and 18.9% and 14.4% of patients had *FLT3-ITD* and *TP53* mutations, respectively [6]. Several previous research indicated that patients with the above adverse molecular or clinical risk factors may not respond well to VEN + HMA treatment [5,6,22,23].

The prognosis for R/R AML remains poor, with a 2-year OS rate between 9% and 20% [1,24]. Allo-HSCT represents the only curative approach for R/R AML, improving the post-transplant 2-year OS ranging from 25% to 58% [25,26,27,28]. In our study, the 2-year OS was 40.2% for overall R/R AML patients and 59.6% for R/R AML patients receiving allo-HSCT, respectively, which was higher than previous studies [1,24,25,26,27,28]. The median OS in our patients was 11.5 months, compared to that in previous reports ranging from 4.0 to 10.7 months [8,9,10,11,14,21]. The improvement of OS in our study could be attributed to the following reasons. First, the median age of our patients is 47 years, which is younger than that reported in other studies, ranging from 59 to 74 years [6,10,29]. Second, a higher proportion (37.1%) of our patients undergoing sequential transplantation after VEN therapy was higher than that in previous studies, ranging from 9.8% to 29.0% [8,9,10,11,14,21]. In addition, most transplant patients (89.0%) in our study received haplo-HSCT, which was associated with a stronger graft-versus-leukemia (GVL) effect [30,31].

Allo-HSCT is recommended to improve the survival of R/R AML patients better regardless of whether they achieve CRc after induction therapy. Interestingly, we found that among patients achieving CRc, the impact of allo-HSCT on OS varied significantly based on MRD status. For MRD-negative patients, OS was comparable between those who underwent allo-HSCT and those who did not (*p* = 0.15), suggesting that achieving MRD negativity is a strong prognostic indicator that may reduce the necessity of allo-HSCT in this subgroup. The favorable outcomes observed in MRD-negative patients without allo-HSCT indicate that allo-HSCT may provide limited additional benefit for these patients. In contrast, MRD-positive patients who underwent allo-HSCT demonstrated significantly prolonged OS compared to those who did not (*p* = 0.003). This highlights the critical role of allo-HSCT in mitigating the poor prognosis associated with MRD positivity, likely through the GVL effect, which helps eradicate residual disease and improve long-term survival outcomes. Therefore, based on our findings, it is recommended that R/R AML patients achieve CRc after induction therapy but remain MRD-positive and undergo allo-HSCT to improve their OS.

Our study identifies significant associations between specific molecular characteristics and treatment efficacy as well as OS in R/R AML patients treated with VEN + HMAs. A novel finding in our study is that *GATA2* mutation was associated with poor treatment responses as well as inferior OS in these patients. De novo AML with *GATA2* mutations was classified in the high-risk group according to the 2022 ELN risk stratification [16]. A previous study has indicated that *GATA2* mutation is associated with a greater risk of cumulative incidence of relapse (CIR) or shorter leukemia-free survival (LFS) in newly diagnosed AML patients treated with a combination of decitabine, cytarabine, aclarubicin, and G-CSF (DCAG) [32]. However, studies about the treatment response of R/R AML patients with *GATA2* mutation to the VEN + HMAs regimen were scarce. Large-scale, prospective, randomized controlled studies are needed to confirm our findings. Regarding the negative impact of *FLT3-ITD* mutation and *DNMT3A* mutation on OS, our results align with the Wang et al. reports [33]. Although the proportion of *FLT3-ITD* mutation (40.1%) in our study was higher than that (12.0–22.8%) in other studies [14,29,34], the OS of our patients was comparable with that reported before. It is noteworthy that 44.3% of patients with *FLT3-ITD* mutations in our study underwent allo-HSCT after VEN + HMAs treatment, which could greatly improve the prognosis of these patients. Additionally, 18 (9.1%) patients received *FLT3-ITD* inhibitors concurrently from the induction therapy stage and continued during long-term maintenance. Our previous study indicated that combining *FLT3* inhibitors (gilteritinib) with VEN + AZA in treating *FLT3mut* R/R AML patients was potentially associated with longer OS [35]. A previous phase 3 trial demonstrated that *FLT3-ITD* inhibitors (sorafenib) significantly prolonged OS and reduced relapse rates when used as maintenance therapy following allo-HSCT in patients with *FLT3* mutations [36]. We also identified a novel association, indicating that R/R AML patients with *NPM1* mutations tend to have improved OS. *NPM1* mutations had previously been reported to predict better response to VEN + HMAs; however, no OS advantage has been reported despite an increased response to VEN + HMAs [6,10,14]. Abaza Y. et al. have reported that in elderly patients with newly diagnosed AML treated with VEN + HMAs as frontline therapy, *NPM1* mutations are associated with higher response rates as well as longer OS [37]. The consistency of *NPM1* mutations in improving OS and treatment response for R/R AML patients treated with VEN + HMAs requires further investigation. *SRSF2* mutations in our study exhibited improved responses to the VEN + HMAs treatment regimen was consistent with the report by Wang et al. [33]. Unlike the findings reported previously [3,10,14,38], our study did not observe a significant improvement in treatment responses in overall R/R AML patients with *IDH1/2* mutations. Notably, in our study, 13.5% of *IDH*-mutated R/R AML patients had participated in clinical trials for *IDH* inhibitors prior to receiving VEN + HMA therapy. These patients only began treatment with VEN + HMAs after failing to respond to the *IDH* inhibitors. Among *IDH*-mutated patients, those who had previously received *IDH* inhibitors demonstrated a comparable response to VEN + HMAs compared to those who had not (CRc, 60.0% vs. 54.7%, *p* = 0.63). Prior exposure to *IDH* inhibitors might not influence the subsequent response of *IDH*-mutated patients to VEN + HMAs therapy.

Our study has unveiled a groundbreaking finding: the *CBFB-MYH11* fusion gene appears to be associated with significantly better OS in the R/R AML patients treated with VEN + HMAs. Consequently, further studies are needed to validate this finding. In our study, the *RUNX1::RUNX1T1* fusion gene showed no clear association with response rates or OS in patients treated with VEN + HMAs. A previous study demonstrates that newly diagnosed *RUNX1::RUNX1T1* AML patients treated with VEN + HMA achieve a significantly lower CRc rate compared to the standard “7 + 3” regimen (27.8% vs. 61.8%, *p* = 0.02). Similarly, the ORR was also inferior with VEN + HMA (27.8% vs. 64.7%, *p* = 0.001). These findings indicate the poor efficacy of VEN + HMA in this AML subset [39]. Interestingly, this poor efficacy was not observed in our R/R AML cohorts. Further large-scale, prospective studies are needed to confirm these findings in R/R AML.

### Limitations

Our study had some limitations. First, our study was a single-center, retrospective study, so potential patient selection bias was inevitable. The exclusion of patients without NGS data introduces a potential selection bias. The decision to perform NGS was influenced by clinical judgment, resource availability, and patient condition, which may limit the generalizability of the results. This limitation might have influenced the representativeness of the cohort, as patients without molecular data may differ in clinical or biological characteristics. Second, a substantial proportion of patients in our cohort had prior exposure to VEN or HMAs. This variability in treatment intent, combination, and duration across different phases introduces heterogeneity in the cohort. Specifically, patients with prior VEN or HMA exposure may have developed distinct biological or clinical characteristics, potentially influencing their response to VEN + HMA therapy in the relapsed/refractory setting. Third, the follow-up duration for assessing survival outcomes was relatively short. Future research should aim for a prospective design and extended follow-up periods to verify these findings further.

## 5. Conclusions

This study highlights the significant efficacy of VEN + HMAs as a treatment for R/R AML, demonstrating its potential to improve patient outcomes in this challenging population. Moreover, the findings underscore the critical role of genetic profiling in optimizing personalized treatment strategies, as specific genetic mutations were found to influence both response rates and overall survival. This research provides valuable guidance for tailoring therapies and improving clinical decision-making by identifying favorable and unfavorable genetic predictors. Ultimately, these results emphasize the need for an integrated approach that combines innovative treatments like VEN + HMAs with advanced molecular diagnostics to enhance survival and quality of life for R/R AML patients.

## Figures and Tables

**Table 1:** Baseline characteristics of patients.

Parameters	All Patients (*n* = 197)
Gender, *n* (%), male/female	107 (54.3)/90 (45.7)
Age, median (range), years	47 (18, 83)
Disease type, *n* (%)	
Primary refractory	71 (36)
Relapsed	126 (64)
Prior HMA, *n* (%)	111 (56.3)
Prior lines before initiating therapy, *n* (%), ≤2/>2	95 (48.2)/102 (51.8)
Prior transplantation, *n* (%)	30 (15.2)
Cycles of VEN, median (range)	1 (1–5)
HMA type, *n* (%)	
Azacitidine	184 (93.4)
Decitabine	13 (6.6)
Underwent allo-HSCT after VEN therapy, *n* (%)	73 (37.1)
Haplo-HSCT	59 (80.8)
HLA-matched sibling donor (MSD) HSCT	12 (16.4)
HLA-matched unrelated donor (URD) HSCT	2 (2.7)
2022 ELN risk stratification, *n* (%)	
Favorable	38 (19.3)
Intermediate	51 (25.9)
Adverse	108 (54.8)
WBC, median (range), ×10^9^/L	2.5 (0.01, 128.00)
HB, median (range), g/L	83.0 (44.0, 164.0)
PLT, median (range), ×10^9^/L	45.0 (1.0, 814.0)
Bone marrow blast, median (range), %	22.0 (2.0, 86.0)
Peripheral blood blast, median (range), %	18.0 (1.0, 85.0)
FAB type, *n* (%)	
M0	1 (0.5)
M1	5 (2.5)
M2	76 (38.6)
M4	34 (17.3)
M5	46 (23.4)
M6	2 (1.0)
M7	1 (0.5)
Missing	32 (16.2)
Type of AML at initial diagnosis, *n* (%)	
De novo AML	168 (85.3)
Secondary AML	26 (13.2)
Therapy-related AML	3 (1.5)
Common molecular mutation, *n* (%)	
NPM1	81 (41.1)
FLT3-ITD	79 (40.1)
RAS(NRAS + KRAS)	75 (38.1)
TP53	60 (30.5)
ASXL1	56 (28.4)
RUNX1	49 (24.9)
DNMT3A	47 (23.9)
TET2	44 (22.3)
SF3B1	44 (22.3)
EVI1	38 (19.3)
EZH2	37 (18.8)
biCEBPA	37 (18.8)
SRSF2	32 (16.2)
IDH2	28 (14.2)
IDH1	24 (12.2)
GATA2	22 (11.2)
Fusion gene, *n* (%)	
RUNX1-RUNX1T1	59 (29.9)
CBFB-MYH11	26 (13.2)
NUP98-NSD1	12 (6.1)
MECOM rearrangement	11 (5.6)
BCR-ABL	7 (3.6)
All-cause mortality, *n* (%)	110 (55.8)

Abbreviations: HMA, hypomethylating agent; VEN, venetoclax; allo-HSCT, allogeneic hematopoietic cell transplantation; Haplo-HSCT, haploidentical HSCT; HLA, human leukocyte antigen; ELN, European Leukemia Net; WBC, white blood cell; HB, hemoglobin; PLT, platelet; FAB, French-American-British; AML, acute myeloid leukemia; FLT3-ITD, internal tandem duplication of the FLT3 gene.

**Table 2:** Outcomes of treatment.

Outcomes	All Patients (*n* = 197)
Responses, *n* (%)	
ORR (CRc + PR)	118 (59.9)
CRc (CR + CRi)	88 (44.7)
CR	39 (19.8)
CRi	49 (24.9)
PR	30 (15.2)
NR	79 (40.1)
Relapse	
Relapse rate of patients with CRc, *n* (%)	33 (37.5)
Duration of response, median (range), months	5.0 (0.6, 20.0)
Survival	
Overall survival, median (range), months	11.5 (0.4, 52.6)
Follow-up, median (range), months	14.0 (0.7, 54.0)

Abbreviations: ORR, overall response rate; CRc, composite complete remission; PR, partial remission; CR, complete remission; CRi, CR with incomplete hematological recovery; NR, non-remission.

## Data Availability

The raw data supporting the conclusions of this article will be made available by the authors, without undue reservation.

## Supplementary Materials

The following supporting information can be downloaded at: https://www.mdpi.com/article/10.3390/cancers17040586/s1, Table S1. Next-generation sequencing (NGS) for 139 genes associated with myeloid tumors; Table S2. Univariable analysis for risk factors of response (CRc); Table S3. Univariate analysis for the risk factors of overall survival (OS).
