# Supplementary material for: Clinical and Molecular Predictors of Response and Survival Following Venetoclax Plus Hypomethylating Agents in Relapsed/Refractory Acute Myeloid Leukemia: A Single-Center Study in Chinese Patients
Source: Cancers (Basel). 2025 Feb 8;17(4):586. doi: 10.3390/cancers17040586 (PMC11852425; doi:10.3390/cancers17040586)
Supplement: Supplementary file 1 [file cancers-17-00586-s001.zip › Supplementary Table S1. The 139-gene NGS panel..pdf]

Table S1. Next-generation sequencing (NGS) for 139 genes associated with myeloid tumors.

ANKRD26	*ABCB1*	*ABL1*	*AKT3*	*ARID1A*	*ARID1B*	*ARID2*	*ASXL1*
ASXL2	*ATG2B*	*ATM*	*ATRX*	*BCL2*	*BCL6*	*BCOR*	*BCORL1*
BLM	*BPGM*	*BRAF*	*BRCA1*	*BRCA2*	*BRIP1*	*CALR*	*CBL*
CBLB	*CBLC*	*CCND3*	*CDKN1A*	*CDKN2A*	*CDKN2B*	*CEBPA*	*CHEK2*
CREBBP	*CRLF2*	*CSF1R*	*CSF3R*	*CTCF*	*CUX1*	*DDX41*	*DIS3*
DKC1	*DNMT3A*	*EED*	*EGFR*	*EGLN1*	*ELANE*	*EP300*	*EPOR*
ERG	*ETNK1*	*ETV6*	*EZH2*	*FBXW7*	*FGFR3*	*FLT3*	*G6PC3*
GATA1	*GATA2*	*GATA3*	*GFI1*	*GNAS*	*GNB1*	*GSKIP*	*HAX1*
HRAS	*ID3*	*IDH1*	*IDH2*	*IKZF1*	*IKZF2*	*IKZF3*	*IL7R*
JAK1	*JAK2*	*JAK3*	*KDM6A*	*KIT*	*KMT2A*	*KMT2B*	*KMT2C*
KMT2D	*KRAS*	*LMO2*	*MPL*	*MYC*	*NBN*	*NF1*	*NOTCH1*
NOTCH2	*NPM1*	*NRAS*	*NT5C2*	*NTRK1*	*PAX5*	*PDGFRA*	*PDGFRB*
PHF6	*PIGA*	*PML*	*PPM1D*	*PRPF8*	*PTEN*	*PTPN11*	*RAD21*
RARA	*RB1*	*RUNX1*	*SBDS*	*SETBP1*	*SETD2*	*SETDB1*	*SF1*
SF3A1	*SF3B1*	*SH2B3*	*SMC1A*	*SMC3*	*SOCS1*	*SRP72*	*SRSF2*
STAG2	*STAT3*	*STAT5A*	*STAT5B*	*SUZ12*	*TAL1*	*TCF3*	*TERC*
TERT	*TET1*	*TET2*	*TP53*	*TPMT*	*U2AF1*	*U2AF2*	*VHL*
WAS	*WT1*	*ZRSR2*					
