# Supplementary material for: Clinical and Molecular Predictors of Response and Survival Following Venetoclax Plus Hypomethylating Agents in Relapsed/Refractory Acute Myeloid Leukemia: A Single-Center Study in Chinese Patients
Source: Cancers (Basel). 2025 Feb 8;17(4):586. doi: 10.3390/cancers17040586 (PMC11852425; doi:10.3390/cancers17040586)
Supplement: Supplementary file 1 [file cancers-17-00586-s001.zip › Supplementary Table S2. Univariable analysis for risk factors of response (CRc).pdf]

Table S2. Univariable analysis for risk factors of response (CRc).

**Variables**	**Univariable**	
	OR, 95% CI	P-value
Secondary AML at initial diagnosis	0.3 (0.1, 0.9)	0.022
Disease type, relapsed vs refractory	1.1 (0.6, 1.9)	0.831
MECOM rearrangement	0.1 (0.0, 0.9)	0.040
Adverse cytogenetics risk	0.6 (0.4, 1.1)	0.099
Adverse ELN risk stratification	0.5 (0.3, 1.0)	0.036
Prior HMA	0.4 (0.2, 0.7)	0.002
The use of antifungal drugs at the first VEN+HMA cycle	0.6 (0.3, 1.1)	0.086
Early relapse[#]	0.4 (0.2, 0.8)	0.017
K/NRAS	0.6 (0.3, 1.0)	0.056
TP53	0.6 (0.3, 1.1)	0.072
CBFB-MYH11	2.2 (0.9, 5.1)	0.068
IDH1/2	1.8 (1.0, 3.5)	0.062
GATA2	0.2 (0.0, 0.6)	0.005
SRSF2	2.0 (0.9, 4.4)	0.071
NPM1	2.3 (1.3, 4.1)	0.005
U2AF1	0.4 (0.2, 1.1)	0.089

Abbreviations: CRc, composite complete remission; AML, acute myeloid leukemia; ELN, European Leukemia Net; HMA, hypomethylating agent; VEN, venetoclax.

Variables of $P < 0.1$ in univariable analysis were included in the multivariable logistics model.

[#] Early relapse is defined as a patient relapsing within 6 months since the first complete remission (CR1), and late relapse as more than 6 months since CR1.
