# Supplementary material for: Clinical and Molecular Predictors of Response and Survival Following Venetoclax Plus Hypomethylating Agents in Relapsed/Refractory Acute Myeloid Leukemia: A Single-Center Study in Chinese Patients
Source: Cancers (Basel). 2025 Feb 8;17(4):586. doi: 10.3390/cancers17040586 (PMC11852425; doi:10.3390/cancers17040586)
Supplement: Supplementary file 1 [file cancers-17-00586-s001.zip › Supplementary Table S3. Univariate analysis for the risk factors of overall survival.pdf]

Table S3. Univariate analysis for the risk factors of overall survival (OS).

**Variables**	**Univariable**	
	HR, 95% CI	p-value
Gender, Male vs Female	1.5 (1.0, 2.1)	0.055
Secondary AML at initial diagnosis	1.6 (1.0, 2.7)	0.058
Adverse ELN risk stratification	1.5 (1.1, 2.2)	0.025
MECOM rearrangement	2.4 (1.2, 4.7)	0.015
Prior HMA	1.6 (1.1, 2.3)	0.026
Prior VEN	1.4 (1.0, 2.2)	0.073
The cycles of VEN+HMA, >2/≤2	0.6 (0.4, 1.0)	0.030
Allo-HSCT after VEN therapy	0.4 (0.2, 0.6)	<0.001
Early relapse[#]	2.0 (1.2, 3.2)	0.007
NUP98-NSD1	2.0 (1.0, 3.8)	0.041
CBFB-MYH11	0.6 (0.4, 1.0)	0.035
BCR-ABL	2.5 (1.5, 4.1)	0.001
FLT3-ITD	1.5 (1.1 2.2)	0.023
K/NRAS	1.5 (1.0, 2.2)	0.028
NPM1	0.6 (0.4, 0.9)	0.016
TP53	1.7 (1.2, 2.5)	0.006
DNMT3A	1.7 (1.1, 2.5)	0.014
IDH1/2	0.5 (0.3, 0.8)	0.003
SF3B1	1.7 (1.1, 2.5)	0.021
EZH2	1.8 (1.1, 2.8)	0.012
SRSF2	0.5 (0.3, 0.9)	0.032
GATA2	2.3 (1.3, 3.8)	0.002

Abbreviations: AML, acute myeloid leukemia; ELN, European Leukemia Net; HMA, hypomethylating agents; VEN, venetoclax; Allo-HSCT, allogeneic hematopoietic cell transplantation.

[#] Early relapse is defined as a patient relapsing within 6 months since the first complete remission (CR1), and late relapse as more than 6 months since CR1.

Variables of $P < 0.1$ in univariable analysis were included in the multivariable COX regression model.
